# Damage-Associated Molecular Patterns in Mesothelioma: Drivers of Inflammation and Therapeutic Targets

**DOI:** 10.3390/ijms27177859

**Published:** 2026-09-02

**Authors:** Annamaria Molinario, Francesca Caprioglio, Angela A. Rilievo, Marco E. Bianchi, Rosanna Mezzapelle

**Affiliations:** 1 Comprehensive Cancer Center, IRCCS San Raffaele Hospital, 20132 Milan, Italy; molinario.annamaria@hsr.it (A.M.); bianchi.marco@hsr.it (M.E.B.); 2 School of Medicine, Vita-Salute San Raffaele University, 20132 Milan, Italy; caprioglio.francesca@hsr.it (F.C.); rilievo.angela@hsr.it (A.A.R.)

**Keywords:** mesothelioma, inflammation, damage associated molecular patterns

## Abstract

Mesothelioma is a rare and aggressive malignancy arising from mesothelial cells. Despite recent advances in systemic therapies, overall survival remains limited, underscoring the need for a deeper mechanistic understanding of disease pathogenesis and novel therapeutic strategies. In mesothelioma, chronic tissue injury induced by asbestos fibers leads to sustained activation of innate immune pathways and chronic inflammation that actively promote tumorigenesis. The release of Damage-Associated Molecular Patterns (DAMPs)—endogenous molecules that signal cellular stress and damage—contributes to establishing a self-sustaining inflammatory circuit within the pleural microenvironment that promotes tumor initiation and progression and immune evasion. Among DAMPs, High-Mobility Group Box 1 (HMGB1) has emerged as a key regulator of mesothelioma pathogenesis. Several studies demonstrated that mesothelial cells actively secrete HMGB1 in response to asbestos exposure, driving macrophage recruitment, cytokine production, and chronic inflammation. Beyond HMGB1, additional DAMPs—including IL-33, extracellular ATP, cell-free nucleic acids, heat shock proteins, and calreticulin—contribute to inflammasome activation, stromal remodeling, and immune dysregulation. Recent evidence suggests that DAMP signaling in mesothelioma is dysregulated, resulting in chronic inflammation coupled with ineffective antitumor immunity. This review provides a comprehensive synthesis of DAMP biology in mesothelioma, highlighting the emerging therapeutic opportunities targeting DAMP-associated pathways.

## 1. Introduction

Mesothelioma is a rare but highly aggressive cancer originating from mesothelial cells lining the pleura, peritoneum, and, less frequently, the pericardium and tunica vaginalis. The disease is characterized by late diagnosis, rapid progression, and limited therapeutic options. Even with the integration of immune checkpoint blockade into first-line therapy, median overall survival remains approximately 12–18 months, reflecting both intrinsic tumor resistance and the complex biology of the disease [1,2].

The etiological association between asbestos exposure and mesothelioma is well established. However, increasing evidence indicates that asbestos acts not only as a carcinogen but also as a potent inducer of chronic sterile inflammation. Unlike other tumors—driven mostly by a high mutational burden—mesothelioma arises through a prolonged interplay between environmental injury, innate immune activation, and progressive cellular transformation [2,3]. This paradigm has shifted the conceptual framework of mesothelioma toward an inflammation-driven disease, in which persistent immune activation is not a bystander effect but a central driver of tumorigenesis. Chronic inflammatory signaling promotes genomic instability, epigenetic remodeling, and the selection of transformed mesothelial cells capable of surviving under conditions of continuous stress [3,4].

Upon inhalation, asbestos fibers deposit within the pleural cavity, where they persist for decades due to their resistance to biodegradation. Macrophages attempting to clear these fibers undergo a process known as “frustrated phagocytosis”, resulting in sustained production of reactive oxygen species (ROS), pro-inflammatory cytokines, and proteolytic enzymes [5]. This persistent inflammatory state leads to repeated cycles of cell death and regeneration. Importantly, asbestos-induced cell death is predominantly non-apoptotic, favoring the release of intracellular molecules into the extracellular space [6,7]. These molecules function as endogenous danger signals, triggering innate immune responses in the absence of infection.

Damage-Associated Molecular Patterns (DAMPs) represent the key molecular link between asbestos-induced injury and chronic inflammation. These endogenous molecules are released or exposed upon cellular stress, injury, or death and are recognized by pattern-recognition receptors (PRRs) expressed on immune and non-immune cells [8,9,10]. Activation of PRRs by DAMPs triggers downstream signaling pathways, including NF-κB activation, MAPK signaling, and inflammasome assembly, leading to the production of pro-inflammatory cytokines and chemokines. In mesothelioma, persistent DAMP release establishes a feed-forward inflammatory loop that sustains immune activation and simultaneously promotes tumor cell survival and immune evasion [11]. Several works demonstrated that mesothelial cells actively secrete High-Mobility Group Box 1 (HMGB1) protein following asbestos exposure and that extracellular HMGB1 is required for macrophage recruitment and cytokine production, thereby initiating and sustaining the inflammatory microenvironment that precedes tumor development [7,12,13,14]. These findings fundamentally reshaped our understanding of mesothelioma pathogenesis, identifying DAMP signaling as a central driver rather than a secondary consequence of disease.

## 2. Mesothelioma: Epidemiology, Pathogenesis, and Tumor Biology

### 2.1. Epidemiology and Global Burden

Mesothelioma remains a significant global health burden, with an estimated 30,000–40,000 deaths annually worldwide [2]. The incidence varies geographically, reflecting historical patterns of asbestos use, with particularly high rates observed in industrialized regions such as Europe, Australia, and North America [15]. Despite regulatory bans in many countries, mesothelioma incidence continues to rise or plateau due to the long latency period between exposure and disease onset, often 30–50 years [16]. This delayed manifestation represents a major challenge for public health systems and underscores the ongoing impact of past asbestos exposure.

### 2.2. Histological Subtypes and Heterogeneity

Mesothelioma is classified into three main histological subtypes: epithelioid, sarcomatoid, and biphasic. These subtypes differ significantly in morphology, biological behavior, and clinical outcomes. Epithelioid mesothelioma—characterized by round epithelial-like cells—is the most common subtype and is associated with a more favorable prognosis, whereas sarcomatoid mesothelioma—characterized by spindle-like cells—is the rarer and more aggressive subtype, and is more resistant to therapy. Biphasic tumors display features of both subtypes and show intermediate clinical outcomes [17].

While these histological categories remain clinically relevant, recent studies using high-throughput molecular techniques (single-cell RNA-seq and spatial transcriptomic) indicate that pleural mesothelioma heterogeneity is better represented by continuous cellular and molecular states rather than by three fully discrete entities. In particular, tumor-cell programs span an epithelioid-to-sarcomatoid/EMT-related continuum that is accompanied by changes in immune and stromal composition [18]. Multiomic classifications based on transcriptional, epigenetic, and genomic features further identify molecular differences that cut across conventional histological subtypes [18,19]. Mesothelioma heterogeneity extends beyond histology and includes differences in molecular alterations, immune infiltration, and stromal composition, all of which influence disease progression and response to therapy.

### 2.3. Asbestos-Driven Chronic Injury

Asbestos fibers consist of long and thin chains of silicate tetrahedra arranged in highly stable and durable crystal lattices, which confer remarkable resistance to heat, chemical degradation, and mechanical stress. Due to these physicochemical properties, asbestos was extensively used in industry [20]. The pathogenicity of asbestos is primarily linked to its fibrous morphology: inhaled fibers are inherently persistent, and their length and stiffness impair effective phagocytosis and clearance by alveolar macrophages. Consequently, asbestos fibers accumulate within the lung and pleural tissues, inducing chronic inflammation, oxidative stress, and persistent tissue damage that can ultimately lead to fibrosis and malignant diseases such as mesothelioma and lung cancer [3].

Macrophages attempting to engulf these fibers generate large amounts of ROS and inflammatory mediators, leading to oxidative DNA damage and activation of stress-response pathways [21]. At the same time, mesothelial cells respond to injury by releasing cytokines and DAMPs, further amplifying the inflammatory response [14].

### 2.4. Molecular Alterations and Inflammation

Mesothelioma is characterized by a relatively low tumor mutational burden compared to other solid tumors but it exhibits recurrent loss-of-function alterations in tumor suppressor genes, including *BAP1* (BRCA1-associated protein 1), *CDKN2A* (Cyclin-Dependent Kinase Inhibitor 2A), *NF2* (Neurofibromin 2), and *LATS2* (Large Tumor Suppressor Kinase 2) [22,23,24].

Mesothelioma therefore represents a paradigmatic example of the interplay between genetic alterations and chronic inflammation, where these two components reinforce each other in driving tumor initiation and progression [4]. Chronic activation of pro-inflammatory pathways, particularly NF-κB signaling, promotes survival of genetically damaged cells by inhibiting apoptosis and sustaining proliferation, while activation of the NLRP3 inflammasome drives a pro-tumorigenic microenvironment [25,26,27,28].

### 2.5. Tumor Microenvironment: Inflammation and Immunosuppression

The mesothelioma tumor microenvironment (TME) is highly complex and characterized by the coexistence of inflammatory and immunosuppressive signals. Key cellular components include tumor-associated macrophages (TAMs) [29], cancer-associated fibroblasts (CAFs) [30], cancer-derived fibroblast (CDFs) [31], T lymphocytes, and endothelial cells [19,32]. TAMs represent one of the dominant immune populations and are often polarized toward pro-tumorigenic phenotypes that promote tumor growth, angiogenesis, and immune suppression [29].

DAMP signaling plays a central role in shaping the TME promoting macrophage recruitment and activation through receptors such as the receptor for advanced glycation end products (RAGE) and Toll-like receptors (TLRs), leading to sustained production of cytokines and chemokines [33]. At the same time, chronic DAMP exposure contributes to immune exhaustion and the establishment of an immunosuppressive microenvironment, limiting effective antitumor immunity.

## 3. Damage-Associated Molecular Patterns

### 3.1. The Conceptual Evolution of Danger Signaling

The identification of Damage-Associated Molecular Patterns (DAMPs) marked a transformative shift in immunological theory. For decades, the field was governed by the Self–Non-Self Theory [34], which proposed that the immune system’s primary role was the detection of foreign antigens. However, this framework failed to explain the robust immune activation observed in sterile trauma or cancer. In 1994, Polly Matzinger introduced the Danger Theory, asserting that the immune system responds to “danger” rather than “strangeness”. Matzinger hypothesized that cells under stress or undergoing non-programmed cell death release endogenous “danger signals” that provide the necessary co-stimulation for immune activation [35]. This conceptual leap was empirically validated with the discovery of HMGB1 as a cytokine-like mediator [36,37]. In 2004, the term DAMPs was formally adopted to parallel the PAMPs (Pathogen-Associated Molecular Patterns) found in microbes, providing a unified biochemical language for tissue injury and infection [38].

Nowadays, DAMPs are considered endogenous molecules—originating from host cells—acting as a signal of tissue damage to the immune system, thus mediating sterile inflammation [39].

The word “alarmin” was coined by Oppenheim and Yang [40] and popularized by Bianchi [41] to indicate molecules that are released after tissue damage or stress, recruit and activate immune cells, and function as endogenous danger mediators. Alarmin is now largely used interchangeably with DAMP.

### 3.2. Diversity and Classification of DAMPs

DAMPs comprise a heterogeneous group of endogenous molecules, classified according to their subcellular origin, biochemical properties, or functional dynamics (extracellular or nuclear; constitutive or inducible) [39].

Nuclear-derived DAMPs include proteins and nucleic acids normally involved in chromatin organization and genomic maintenance. One of the best-characterized alarmins in mesothelioma is HMGB1, which acts as a potent pro-inflammatory cytokine through different receptors [42,43,44,45]. Similarly, IL-33 acts as a “nuclear alarmin” retained within mesothelial and endothelial cell nuclei until tissue damage occurs [46,47,48], while extracellular nucleic acid can activate innate immune pathways including cGAS–STING and TLR9 signaling [49].

Cytosolic DAMPs include several stress-responsive molecules involved in inflammatory amplification. S100 proteins, a family of calcium-binding proteins, modulate leukocyte recruitment and cytokine production [50], whereas extracellular ATP functions as a danger signal capable of activating purinergic receptors and inflammasome pathways [51]. Uric acid, a byproduct of purine metabolism, can form monosodium urate crystals that strongly activate the NLRP3 inflammasome, promoting IL-1β maturation and chronic inflammation [52].

Mitochondrial DAMPs (mtDAMPs) include mitochondrial DNA (mtDNA), formyl peptides, and cytochrome c, whose extracellular release reflects mitochondrial dysfunction and metabolic stress [53].

Endoplasmic reticulum (ER)-associated DAMPs contribute to inflammatory signaling. Calreticulin (CALR) can translocate to the plasma membrane during ER stress, functioning as a pro-phagocytic signal that modulates immune recognition [54,55].

Extracellular matrix (ECM)-derived DAMPs are generated during chronic tissue remodeling and fibrosis. Degradation products, e.g., hyaluronan fragments and heparan sulfate, accumulate in chronically injured tissues and contribute to persistent inflammatory activation by engaging TLRs and other PRRs [56,57].

From a functional and kinetic perspective, DAMPs can also be classified as constitutive, when preformed and rapidly released following unscheduled death that entails membrane rupture (e.g., HMGB1) [12], or inducible, when synthesized or actively secreted in response to prolonged cellular stress or sublethal injury (e.g., heat shock proteins-HSPs) [58].

In the context of mesothelioma, chronic asbestos exposure induces persistent cellular damage and death, leading to continuous DAMP release, thus establishing a self-sustaining inflammatory circuit which promotes tumor initiation and progression [7,14].

## 4. DAMPs in Mesothelioma Pathogenesis

### 4.1. Nuclear DAMPs in Mesothelioma

Nuclear DAMPs reside inside the nucleus of unperturbed cells. When cells are stressed or die, these molecules can be released outside the cell where they act as alarmins, shaping inflammation and immunity. This concept is particularly relevant in mesothelioma, since the disease develops in a chronically injured pleural environment, where several nuclear DAMP-related axes have been investigated in cancer biology, biomarkers, and translational studies [59,60] (Table 1).

#### 4.1.1. HMGB1

Among nuclear DAMPs relevant to mesothelioma, HMGB1 is one of the best characterized [12,61]. HMGB1 is a non-histone chromatin-binding protein that controls DNA architecture and transcription, supports chromatin stability and genome maintenance, and can be released during immune activation and tissue injury responses [42].

HMGB1 is composed of 214 amino acids and has a molecular weight of approximately about 25 kDa. It is organized into two positively charged DNA-binding domains, termed BoxA (aa 14–77) and BoxB (aa 101–163), embedded in intrinsically disordered regions, including a negatively charged C-terminal tail (aa 184–214) composed exclusively of aspartic and glutamic acid residues [62,63]. Each domain is composed of three α-helices (helix I–III) connected by two loops (loop I–II), forming the characteristic L-shaped fold that enables HMGB1 to interact with DNA. The acidic C-terminal tail acts as an intramolecular regulatory domain and DNA-binding activity, thereby contributing to the autoregulation of HMGB1 functions [64]. HMGB1 contains two nuclear localization signals (NLSs), allowing HMGB1 to shuttle efficiently between the nucleus and the cytoplasm while maintaining its predominant nuclear localization under physiological conditions [65].

HMGB1 reaches the extracellular space through two distinct mechanisms: passive release from damaged cells and active secretion by stressed or immune-activated cells. Passive release occurs following loss of membrane integrity during all lytic forms of cell death, whereas active secretion requires HMGB1 translocation from the nucleus to the cytoplasm, a process promoted by post-translational modifications such as lysine acetylation or serine phosphorylation that prevent nuclear re-entry and favor extracellular export [65,66,67]. In mesothelioma, extracellular HMGB1 may derive both from asbestos-induced mesothelial cell injury and from active secretion by stressed and/or transformed cells. Thus, HMGB1 acts as a key DAMP linking tissue damage to chronic inflammation and tumor-promoting signaling within the TME [12,61]. HMGB1 trafficking is further regulated by BAP1, which forms a complex with HMGB1 and HDAC1 to control HMGB1 acetylation and localization. Loss of BAP1 function favors HMGB1 hyperacetylation, cytoplasmic accumulation, and extracellular release, providing a link between BAP1 deficiency, enhanced DAMP signaling, and mesothelioma susceptibility and progression. In germline BAP1 mutation carriers, circulating acetylated HMGB1 is elevated and increases further upon mesothelioma development, supporting a link between BAP1 loss and dysregulated HMGB1 secretion. Reduced BAP1 promotes HDAC1 destabilization and degradation, favoring HMGB1 acetylation and its translocation from the nucleus to the cytoplasm and extracellular space, where it may contribute to the chronic inflammatory milieu associated with mesothelioma. Whether this BAP1–HDAC1–HMGB1 axis is similarly relevant in sporadic mesothelioma harboring somatic BAP1 loss remains to be established [59,68].

When HMGB1 is outside the cell, it acts as a prototypical DAMP signal of danger. Its extracellular effects depend on its biochemical form and on the receptors engaged. Its activity is critically regulated by the redox state of its three conserved cysteine residues: Cys23 and Cys45 in BoxA, and Cys106 in BoxB. Changes in the oxidation state of these cysteines alter the conformation of the protein, affecting the orientation of the α-helices and consequently the affinity for binding partners and cell surface receptors [69]. These redox modifications determine the extracellular biological functions of HMGB1. Three major redox isoforms of HMGB1 have been identified: in fully reduced HMGB1, all cysteines are in the thiol (-SH) state, and HMGB1 has chemotactic activity [45]; disulfide HMGB1 contains an intramolecular disulfide bond between Cys23 and Cys45 and promotes the production and release of pro-inflammatory cytokines [44]; and in terminally oxidized HMGB1, cysteine residues become irreversibly oxidized to sulfonates [70,71].

Disulfide HMGB1 has been shown to bind to the receptor for advanced glycation end products (RAGE) and TLR4 and to drive sterile inflammatory programs; in its fully reduced form, HMGB1 can enhance CXCR4-dependent chemotaxis by forming a heterocomplex with CXCL12 (also known as Stromal cell-derived factor 1, SDF-1), itself a homeostatic chemokine that regulates cell trafficking via CXCR4 [42,43,44,45]. The fully oxidized isoform is shown to bind RAGE inducing immune tolerance [70,71].

In cancer, extracellular HMGB1 has been associated with immune activation in some therapy-related contexts, but it can also support tumor-promoting inflammatory programs [42]. HMGB1 released from injured mesothelial tissue has been causally implicated in asbestos-associated mesothelioma development, supporting HMGB1 as a mechanistic link between tissue damage and tumor development [12]. Tumor cells have been shown to secrete HMGB1, which supports mesothelioma progression [61]. More broadly, the mesothelioma-focused translational literature frames HMGB1 as a key axis in asbestos-related carcinogenesis and discusses it as a targetable pathway [59,60].

Overall, HMGB1 is currently one of the most studied DAMPs in mesothelioma, because its secretion is connected to tumor development and disease progression [12,61]. In asbestos-exposed subjects and mesothelioma patients, serum levels of HMGB1 are elevated; however, elevated HMGB1 is common across inflammatory conditions and can become diagnostic/prognostic only within multi-marker strategies for early detection and disease monitoring [72].

#### 4.1.2. IL-33

Among nuclear alarmins, IL-33 has been investigated in the pleural compartment and TME, where it reflects and/or modulates local inflammatory programs [46,47,48]. IL-33 is a member of the IL-1 cytokine family that functions either as nuclear factor contributing to tissue homeostasis and repair or as alarmin when released into the extracellular milieu. Extracellular IL-33 signals through the ST2 (IL1RL1) receptor in association with the IL-1 receptor accessory protein (IL-1RAcP), activating downstream inflammatory pathways. The biological effects of the IL-33/ST2 axis are highly context-dependent and vary across tissues and disease settings [73].

In cancer, IL-33 promotes the accumulation and maturation of ST2^+^ Tregs within the TME, enhancing their immunosuppressive activity [74].

IL-33 has been detected in pleural effusions both in inflammatory and mesothelioma related-conditions [46,47]. More recently, integrated transcriptomic analyses identified IL-33-associated inflammatory programs as contributors to mesothelioma progression [48].

#### 4.1.3. cfDNA, ctDNA and exRNA

DAMP signaling can also be mediated by extracellular nucleic acids. Cell-free DNA (cfDNA) comprises extracellular DNA fragments released into biological fluids, while the tumor-derived fraction is referred to as circulating tumor DNA (ctDNA). cfDNA can activate innate immune responses through nucleic acid-sensing pathways. Self-DNA is recognized by several pattern-recognition receptor systems, including TLR9, the cGAS–STING pathway, and AIM2/AIM2-like receptors (ALRs), thereby promoting inflammatory signaling. Consequently, cfDNA can engage innate immune sensors and amplify inflammatory circuits [49]. Current mesothelioma research has investigated circulating DNA as a translational biomarker and leveraged cfDNA/ctDNA for disease detection, molecular profiling, and treatment monitoring [75,76,77].

In parallel with cfDNA, extracellular RNA (exRNA) and extracellular vesicle (EV)-RNA have emerged as biomarkers in liquid biopsies in mesothelioma [78,79,80,81]. exRNA can function as a pro-inflammatory mediator, promoting leukocyte recruitment and activation and contributing to innate immune responses.

These observations support the concept that exRNA can act as a DAMP, although direct evidence for such activity in mesothelioma needs further investigation [81].

### 4.2. Cytosolic DAMPs in Mesothelioma

Beyond nuclear alarmins and extracellular nucleic acids, cytosolic DAMPs have also been implicated in mesothelioma-associated immune responses. Among these, CALR and HSPs are the best-characterized examples.

#### 4.2.1. Calreticulin

Calreticulin (CALR) is a 46 kDa multifunctional Ca^2+^-binding chaperone primarily localized in the ER, where it regulates calcium homeostasis, protein folding, and quality control through the calreticulin/calnexin cycle [82].

In response to ER stress, oxidative damage, or anticancer therapies, CALR can translocate to the cell surface and/or be released extracellularly, where it functions as a DAMP [83,84]. Surface-exposed CALR serves as an “eat-me” signal recognized by CD91/LRP1 receptors on dendritic cells and macrophages, promoting phagocytosis and facilitating antigen uptake and cross-presentation [55]. This event represents an early and critical hallmark of immunogenic cell death (ICD), largely regulated by PERK–eIF2α-dependent ER stress pathways [85,86]. In cooperation with other DAMPs, including HMGB1 and extracellular ATP, CALR supports dendritic cell maturation, pro-inflammatory cytokine release, and cytotoxic T-cell activation, thereby linking tumor cell stress to adaptive antitumor immunity [84,87,88,89].

In mesothelioma, CALR is exposed on the surface of tumor cells, but it is often insufficient to sustain effective immunogenic signaling [90]. CALR can cooperate with HMGB1 and ATP to promote antigen-presenting cell activation, thus contributing to improved responses to immunotherapy [91]. However, the immunosuppressive milieu characterized by TAMs, regulatory T cells, and myeloid-derived suppressor cells (MDSCs) can attenuate CALR-driven immune activation, limiting its translation into effective antitumor immunity [29]. Consequently, CALR is considered both a marker of therapy-induced ICD and a target to enhance tumor immunogenicity in solid tumors [92].

#### 4.2.2. Heat Shock Proteins

HSPs, particularly HSP70 and HSP90, are highly conserved molecular chaperones that maintain cellular proteostasis by facilitating protein folding, preventing protein aggregation, and supporting protein trafficking and degradation [93,94].

In cancer, HSPs are frequently overexpressed and support tumor adaptation to hypoxia, oxidative stress, and chronic inflammatory conditions. HSP90 stabilizes numerous oncogenic signaling molecules, contributing to the maintenance of signaling pathways associated with proliferation and survival. HSP70 inhibits both intrinsic and extrinsic apoptotic pathways, collectively promoting tumor cell survival, stress tolerance, and therapy resistance, promoting the persistence of genetically damaged mesothelial cells [95,96,97,98].

Following cell stress, death, or therapy-induced damage, HSP70 and HSP90 can be released extracellularly, where they function as DAMPs [99,100]. Extracellular HSPs interact with PRRs, e.g., TLR2, TLR4, and scavenger receptors expressed on dendritic cells and macrophages, activating NF-κB- and MAPK-dependent inflammatory pathways [101]. In addition, HSPs can chaperone tumor-derived peptides, facilitating antigen uptake and cross-presentation, thus linking cellular stress to adaptive immune responses [102].

In mesothelioma, extracellular HSPs activate innate immune signaling pathways and promote cytokine production and immune cell recruitment [58,103]. However, within the chronically inflamed and immunosuppressive TME, these responses may be insufficient to generate effective antitumor immunity.

### 4.3. Inflammasome-Related DAMP Signaling

Inflammasomes are cytosolic multiprotein complexes that function as central sensors of cellular stress and tissue damage [104,105].

Activated by both PAMPs and DAMPs, inflammasomes activate caspase-1 and drive the process of maturation and secretion of the pro-inflammatory cytokines IL-1β and IL-18, as well as the induction of pyroptotic cell death [106].

A wide range of DAMPs can trigger inflammasome activation, including extracellular ATP, uric acid crystals, mitochondrial DNA, ROS, and HMGB1, reflecting cellular stress, metabolic dysfunction, and membrane damage [107]. These signals converge primarily on the NLRP3 inflammasome, which is considered the most broadly responsive inflammasome platform in sterile inflammation.

Importantly, inflammasome activation in mesothelioma is tightly linked to asbestos-induced cellular injury. Asbestos fibers trigger chronic ROS production, mitochondrial dysfunction, and continuous release of DAMPs such as ATP and HMGB1, maintaining NLRP3 activation in both mesothelial and infiltrating immune cells [61,108,109,110,111]. This persistent inflammatory loop not only amplifies cytokine production but also reinforces a tumor-permissive environment which supports cancer initiation and progression.

Overall, inflammasome-related DAMP signaling represents a key mechanistic bridge between chronic sterile inflammation and malignant transformation in mesothelioma, highlighting the NLRP3–IL-1β axis as a central driver of tumor-promoting inflammation and a potential therapeutic target [112].

#### 4.3.1. Interleukin-1α

IL-1α is a pro-inflammatory cytokine acting at the interface between cellular stress, tissue damage, and immune activation [113,114].

Cellular injury, death, or stress cause the release of IL-1α into the extracellular space, where it acts as a DAMP by binding to the IL-1 receptor (IL-1R1) [115] and activating downstream signaling pathways involving NF-κB and MAPKs; this results in enhanced leukocyte recruitment and sustained inflammatory responses [116,117,118].

Mesothelial cells express IL-1α, which is rapidly released following asbestos-induced necrotic cell death, a key event in mesothelioma initiation [119]. IL-1R1 signaling in neighboring cells leads to NF-κB-dependent production of IL-6, IL-8, and other chemokines that promote the recruitment of inflammatory cells into the pleural microenvironment [120]. In chronic inflammatory diseases and cancer, including mesothelioma, continuous IL-1α release maintains a pro-inflammatory microenvironment which promotes fibroblast activation, extracellular matrix remodeling, and recruitment of immunosuppressive myeloid populations, thus supporting tumor progression and immune evasion [116,118,121].

Overall, IL-1α establishes a self-sustaining inflammatory circuit characterized by cycles of cell death and cytokine release, representing a promising therapeutic target to disrupt inflammation-driven mesothelioma progression.

#### 4.3.2. Adenosine Triphosphate

ATP is the primary intracellular energy currency and also functions as an extracellular signaling molecule, particularly when released from stressed or damaged cells; extracellular ATP activates purinergic receptors and modulates immune and inflammatory responses [51].

Tumor cells frequently rely on aerobic glycolysis (the Warburg effect) to support ATP production and diversion of metabolic intermediates into biosynthetic pathways [122]. In parallel, extracellular ATP accumulates in the TME due to hypoxia and cell stress and death, where it functions as a DAMP that regulates immune responses, inflammation, and stromal interactions [123].

In mesothelioma, cell injury induced by asbestos is associated with ATP release into the pleural microenvironment, where it activates purinergic receptors such as P2X7 and P2Y family members [124,125]. P2X7 activation promotes potassium efflux, a signal for NLRP3 inflammasome assembly, leading to IL-1β and IL-18 maturation [126,127]. In addition, extracellular ATP promotes P2X7-dependent Treg accumulation and suppressive activity within the TME, thereby contributing to impaired antitumor T-cell responses and tumor immune evasion [128].

This fosters a chronic inflammatory milieu characterized by immune cell recruitment, fibroblast activation, and tumor-promoting cytokine production; this persistent inflammatory circuit promotes tumor progression and immune evasion [129].

#### 4.3.3. Calcium Ions

Ca^2+^ is a universal second messenger that regulates a broad range of cellular processes, including gene expression, secretion, motility, metabolism, and apoptosis [130].

Dysregulated Ca^2+^ entry and altered channel activity are frequently observed in tumor cells and support oncogenic signaling pathways controlling cell cycle progression and survival [131].

Extracellular Ca^2+^ can also be detected by surface receptors and act as a DAMP; calcium-sensing receptor (CaSR) and G protein-coupled receptor (GPCR) class C group 6 member A (GPRC6A) have been proposed to act as sensors of endogenous danger by integrating extracellular Ca^2+^ fluctuations with tissue damage-associated cues. Through Ca^2+^ mobilization, GPRC6A can promote NLRP3 inflammasome activation linking calcium signaling to sterile inflammation and tumor-promoting immune responses [132].

In mesothelioma, Ca^2+^ signaling is altered and contributes to multiple aspects of tumor biology. Aberrant expression of Ca^2+^ channels, exchangers, and pumps leads to sustained intracellular Ca^2+^ fluxes, which activate oncogenic pathways controlling proliferation and resistance to apoptosis [133,134]. These adaptations allow mesothelioma cells to survive in the hypoxic, nutrient-limited pleural environment. In addition, Ca^2+^ signaling interfaces with inflammatory pathways, regulating cytokine secretion, inflammasome activation, and stromal remodeling within the TME [135,136]. Overall, dysregulated Ca^2+^ homeostasis acts as an integrative node linking metabolism, inflammation, and tumor–stroma communication.

#### 4.3.4. Reactive Oxygen Species

ROS are highly reactive oxygen-derived molecules generated as natural products of cellular metabolism. ROS function as essential signaling mediators regulating cell proliferation, differentiation, immune responses, and adaptation to stress [137,138,139]. However, excessive ROS levels are cytotoxic: ROS can induce DNA damage and lipid peroxidation that can lead to cell death via ferroptosis. In cancer, ROS levels are typically increased due to oncogenic signaling, mitochondrial dysfunction, hypoxia, and metabolic reprogramming [140]. This moderate elevation in ROS contributes to tumor progression by activating redox-sensitive signaling pathways such as MAPK [141], p53 [142], PI3K/AKT [143], NRF2 [144], JAK/STAT [145], TGF-β [146], HIF1α [147], and NF-κB [148], which promote proliferation, survival, and inflammatory responses.

In mesothelioma, asbestos fibers induce persistent ROS generation [149] driving malignant transformation in the pleura [3]. High intracellular ROS levels cause DNA damage in mesothelioma cells and trigger the activation of antioxidant response pathways representing promising biomarkers for mesothelioma [150].

Elevated ROS also interact with DAMP signaling pathways, including HMGB1 and ATP release, further enhancing sterile inflammation and immune dysregulation [151].

**Table 1:** DAMP–receptor signaling pathways and their biological functions in mesothelioma.

Biochemical Class	DAMP	Receptor	Downstream Pathway	Biological Effect in Mesothelioma
Protein	HMGB1	TLR4, RAGE	MyD88 → NF-κB, MAPK/ERK, PI3K/AKT	Chronic inflammation [37], cytokine production [42], macrophage recruitment [43]; enhanced tumor cell survival, proliferation [61], invasion, autophagy, and resistance to cell death
	HSP70, HSP90	TLR4, CD91	MyD88 → NF-κB; antigen-processing pathways	Inflammatory signaling and immune modulation within the tumor microenvironment [101,102,103]
	S100 proteins	RAGE	MAPK/ERK, PI3K/AKT	Inflammation, tumor progression and metastasis formation [50,152,153]
	IL-33	ST2	MyD88 → NF-κB, MAPK, Th2 cytokines	Fibrosis, pro-tumoral macrophage phenotype, immunosuppressive microenvironment [154,155,156]
	CALR	CD91	Phagocytosis and antigen presentation pathways	Induction of ICD, although often subverted in mesothelioma [55,90]
Nucleotide	ATP	P2X7	NLRP3 inflammasome → Caspase-1	IL-1β maturation, chronic inflammation, recruitment of MDSCs [51,125,126]
Nucleic Acid	cfDNA	TLR9	MyD88 → IRF7/NF-κB	Inflammatory responses and tumor-associated immune activation [149]
	mtDNA	TLR9, cGAS-STING	STING → TBK1 → IRF3	Type I interferon signaling, inflammation, modulation of antitumor immunity [49]
Ion	Ca^2+^	CaSR, GPRC6A	Calcium-dependent signaling cascades	Regulation of proliferation, inflammation, and cell survival [132,134,135]

### 4.4. DAMP-Associated Signaling Modulators

**Figure 1:**
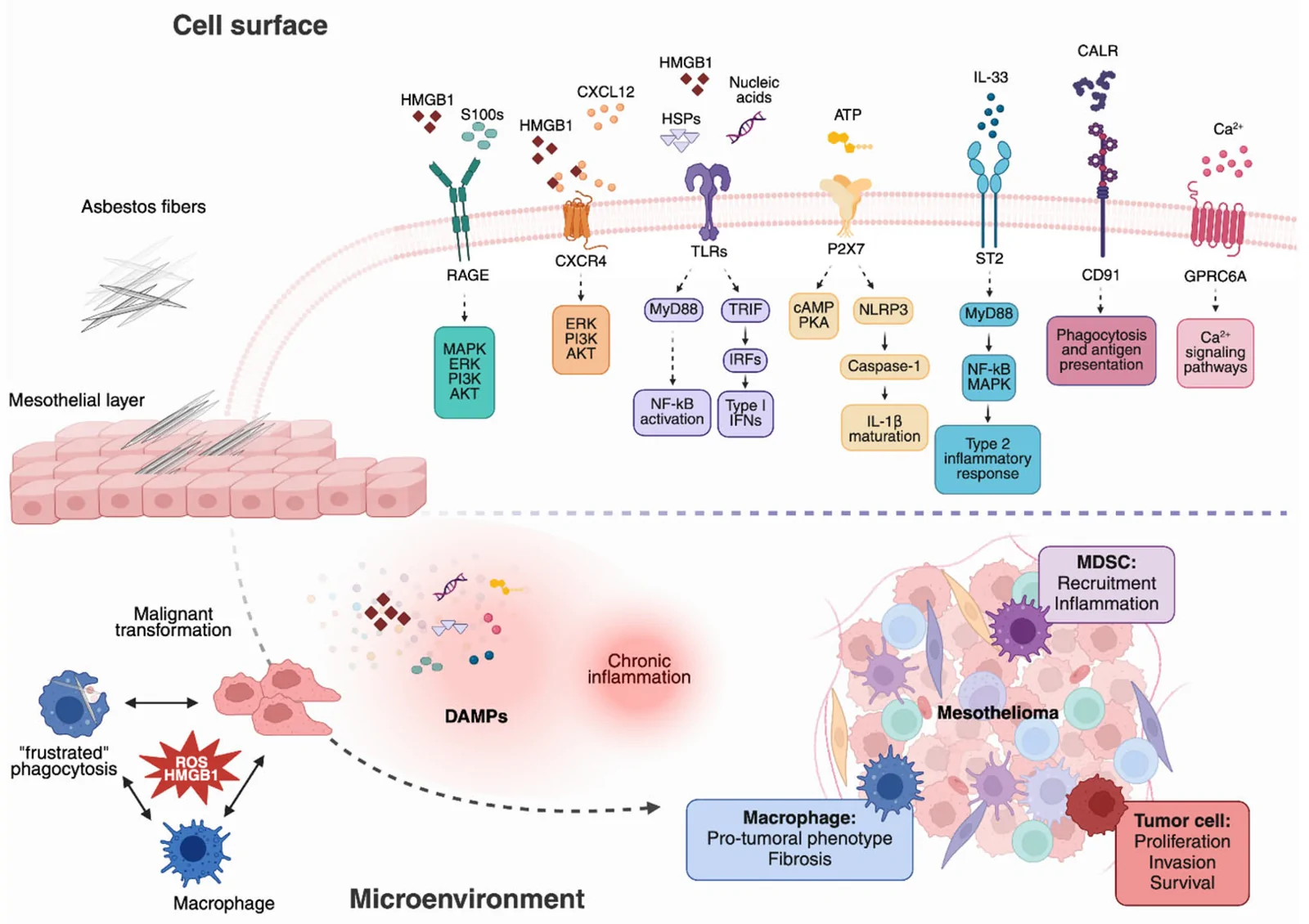
DAMP-mediated signaling pathways and their role in mesothelioma. (**Upper**) Upon asbestos-related tissue injury, DAMPs—including HMGB1, ATP, nucleic acids, heat shock proteins (HSPs), S100 proteins, IL-33, calreticulin (CALR), and calcium—are released extracellularly, where they interact with cognate receptors. The activation of RAGE, CXCR4, Toll-like receptors (TLRs), P2X7, ST2, CD91, and GPRC6A triggers multiple downstream signaling pathways, including MAPK, ERK, PI3K/AKT, NF-κB, MyD88, TRIF/IRFs, and the NLRP3 inflammasome, leading to inflammatory cytokine production, type I interferon responses, IL-1β maturation, and calcium-dependent signaling. Recognition of surface-exposed CALR by CD91 further promotes phagocytosis and antigen presentation. (**Lower**) Asbestos fibers induce persistent mesothelial injury and frustrated phagocytosis by macrophages, resulting in the production of reactive oxygen species (ROS), HMGB1, and other DAMPs, contributing to malignant transformation and chronic inflammation. The continuous release of DAMPs into the tumor microenvironment shapes immune and stromal responses. DAMP-mediated signaling promotes the recruitment of myeloid-derived suppressor cells (MDSCs), the polarization of macrophages toward pro-tumoral and pro-fibrotic phenotypes, and supports tumor cell proliferation, invasion, and survival. Created in BioRender. Caprioglio, F. (2026) https://BioRender.com/zluw44z (accessed on 30 August 2026).

The biological activity of DAMPs is highly context-dependent and influenced by post-translational modifications, redox state, and PRR availability. A single DAMP may engage multiple receptors and activate distinct downstream pathways. HMGB1 exemplifies this signaling promiscuity, as different redox isoforms can interact with different receptors [157]. Moreover, DAMP signaling also dynamically shapes interactions between tumor cells, stromal components, and infiltrating immune cells, thereby contributing to mesothelioma progression and therapeutic resistance [8,158] (Figure 1). In the contest of mesothelioma, the most well-characterized PRRs are TLRs, RAGE, NOD-like receptors (NLRs), P2X7, and cGAS–STING [152]. Activation of these receptors triggers intracellular signaling pathways involving NF-κB, MAPKs, IRF3, and inflammasome complexes, resulting in the production of pro-inflammatory cytokines, chemokines, and type I interferons that sustain chronic inflammation and tumor progression [159,160,161,162].

TLR2 and TLR4 are major sensors of HMGB1, HSP70, HSP90, and S100 proteins released following asbestos-induced cellular damage [152]. Their activation induces MyD88-dependent signaling cascades leading to NF-κB activation and the expression of inflammatory mediators such as TNF-α and IL-6, as well as anti-apoptotic proteins, i.e., BCL-xL, which promote the survival of damaged mesothelial cells [14,163,164,165]. TLR9 can also recognize DNA released following cell death, contributing to inflammasome activation and cytokine production [166,167].

RAGE, encoded by the *Ager* gene, is a multiligand PRR belonging to the immunoglobulin superfamily that signals through interaction with diaphanous-related formin-1 (DIAPH1) [168]. In mesothelioma, RAGE represents one of the most important signaling hubs due to its ability to bind multiple DAMPs, including HMGB1, S100 proteins, phosphatidylserine, nucleic acids, and advanced glycation end products (AGEs) [153]. AGEs are stable adducts formed through non-enzymatic glycation and oxidation of proteins, lipids, and nucleic acids; they accumulate progressively within tissues and ECM components, where they alter matrix architecture, increase tissue stiffness, and promote fibrotic remodeling [169,170]. Upon ligand engagement, RAGE activates NF-κB, MAPK, PI3K/Akt, STAT3, and Src-family kinase pathways, thereby promoting inflammatory cytokine production, tumor cell survival, migration, invasion, and metabolic adaptation [7,171,172]. Specifically, the HMGB1–RAGE axis plays a central role: mesothelioma cells expressing elevated HMGB1 levels also display increased RAGE expression, creating a positive feedback loop sustaining tumor cell proliferation and viability [61]. Concomitantly, AGE formation is a consequence of asbestos-induced tissue injury [173].

Overall, persistent activation of DAMP–PRR signaling pathways establishes a self-sustaining inflammatory circuit that promotes mesothelioma initiation, progression, immune evasion, and therapeutic resistance.

## 5. Clinical and Translational Implications

### 5.1. DAMPs as Biomarkers in Mesothelioma

DAMPs have emerged as promising biomarker candidates in mesothelioma because they reflect ongoing tissue injury, inflammation, and tumor–host interactions, and thus are mechanistically related to mesothelial transformation and disease progression [7,14,61,174]. Unfortunately, no DAMP is a validated predictor or indicator for the progression from asbestos-related tissue injury to mesothelioma.

Among DAMP-related biomarkers, HMGB1, extracellular ATP, cfDNA/RNA, mtDNA, and HSPs are generally associated with increased tumor burden, persistent tissue damage, and more aggressive disease phenotypes [175,176,177] (Table 2).

HMGB1 is actively and passively secreted in response to asbestos-induced cell stress and death and is mechanistically linked to mesothelioma progression through activation of RAGE- and TLR-dependent signaling pathways, which sustain NF-κB activation and inflammasome priming [178,179]. Elevated circulating and pleural HMGB1 levels have been associated with asbestos exposure, mesothelioma development, disease stage, and clinical outcome, highlighting its potential utility for risk stratification and prognosis [180,181]. Nevertheless, circulating HMGB1 has limited disease specificity because it is elevated in numerous inflammatory, malignant, and necrotic conditions. Greater diagnostic potential may therefore reside in the characterization of specific molecular forms of HMGB1, including acetylated HMGB1, and in its integration with additional circulating biomarkers rather than its use as an isolated marker [180].

Extracellular ATP, frequently detected in pleural effusions, reflects ongoing cellular stress and tissue damage and is linked to the P2X7–NLRP3 inflammasome axis [182]. Although its clinical utility remains incompletely defined, ATP-related signaling components may provide insight into the inflammatory status of TME.

cfDNA/ctDNA have emerged as promising liquid biopsy biomarkers in mesothelioma. While cfDNA originates from both normal and malignant cells undergoing apoptosis or necrosis, ctDNA represents the tumor-derived fraction carrying cancer-specific genetic alterations [183,184]. Recent studies have demonstrated the feasibility of personalized ctDNA monitoring based on patient-specific chromosomal Copy Number Variations, supporting its potential use for disease tracking, minimal residual disease assessment, and treatment monitoring [75]. Moreover, ctDNA analysis has been incorporated as a translational endpoint in perioperative immunotherapy trials for diffuse pleural mesothelioma, highlighting its growing relevance in clinical research [76]. These approaches are especially attractive because repeated blood sampling may provide dynamic information that cannot be obtained from a single tissue biopsy. However, the relatively low abundance of ctDNA and the limited number of recurrent somatic variants in mesothelioma remain important technical challenges, supporting the development of individualized or tumor-informed approaches [75,184].

Extracellular RNA species have similarly attracted interest as liquid biopsy biomarkers in mesothelioma. Circulating miRNA signatures have been characterized in mesothelioma patients and show promise for disease detection and monitoring [78,80]. In parallel, EV-RNA cargo has been investigated to characterize vesicle composition and identify clinically relevant biomarkers [79]. More recently, the range of EV-RNA species explored in mesothelioma has expanded to include circular RNAs and other small RNA populations, further supporting the potential of EV-based liquid biopsy approaches [185].

Additionally, mtDNA fragments are increasingly recognized as indicators of inflammatory activity and disease burden. Extracellular mtDNA can activate innate immune pathways such as TLR9 and cGAS–STING, contributing to chronic inflammation and maintenance of a pro-tumorigenic microenvironment [175]. Elevated circulating mtDNA levels have been associated with adverse outcomes in several inflammatory and malignant conditions, suggesting their potential prognostic relevance in mesothelioma. However, direct clinical evidence supporting circulating mtDNA as a diagnostic or prognostic biomarker in mesothelioma remains limited. Its translational value in this disease therefore requires dedicated prospective investigation rather than extrapolation from other malignancies.

HSPs represent another potential biomarker class, but most studies have focused on therapeutic targeting in mesothelioma rather than biomarker development [186]. Altered expression patterns of HSP90 isoforms, including increased levels of the mitochondrial homolog TRAP1, have been reported in lung cancer and suggest the existence of tumor-specific chaperone signatures [187]. Likewise, dysregulation of the HSP70 chaperone network has been associated with malignant transformation and altered proteostasis [187].

Moreover, systemic biochemical alterations may also provide prognostic information in mesothelioma, e.g., serum calcium levels have been identified as an independent predictor of poor survival, suggesting that disruption of metabolic and inflammatory homeostasis may reflect disease severity and clinical outcome [188].

Overall, circulating DAMPs are promising candidates for liquid-biopsy strategies in mesothelioma, particularly for longitudinal disease monitoring and in combination with tumor-specific genomic, inflammatory, and immune biomarkers. Clinical translation will require validation in prospective cohorts, appropriate control groups, and standardized pre-analytical procedures, especially for HMGB1, whose levels and biological activity are strongly influenced by sample handling, post-translational modifications, and redox state [189,190].

**Table 2:** Clinical relevance of major DAMPs and DAMP-associated biomarkers in mesothelioma.

Category	DAMP	Clinical Utility and Significance in Mesothelioma
Predictive	CALR	Surface CALR expression may identify tumors where effective antitumor immune responses occur after chemotherapy or radiotherapy [90,91]. Potential biomarker of ICD induction in mesothelioma [92].
	Extracellular ATP	Reflects treatment-induced ICD and local immune activation [51]. The ATP/adenosine balance within the tumor microenvironment may predict response to chemotherapy, radiotherapy, and immunotherapy [124,125].
Prognostic	IL-33	Increased IL-33/ST2 signaling has been associated with a pro-tumorigenic and immunosuppressive microenvironment [46], potentially contributing to mesothelioma progression [48].
	S100 Proteins	Elevated expression may correlate with inflammatory burden and disease progression [50,152,153], although clinical validation in mesothelioma remains limited.
Diagnostic, Monitoring	cfDNA/mtDNA	Emerging liquid-biopsy biomarkers potentially useful for disease detection and monitoring treatment response in mesothelioma [75,76,183,184].
Diagnostic, Prognostic, Predictive	HMGB1	Elevated circulating HMGB1, particularly hyperacetylated HMGB1, distinguishes mesothelioma patients from asbestos-exposed individuals and is associated with disease progression and poor outcome [174,181]. HMGB1 may also predict responsiveness to therapies inducing ICD.
Prognostic, Monitoring	HSP70, HSP90	Increased HSP expression is associated with tumor aggressiveness, treatment resistance, and disease progression [187]. Circulating HSPs are being explored as minimally invasive biomarkers for disease monitoring.

### 5.2. Therapeutic Targeting of DAMP Signaling

In cancer, DAMP-targeted interventions generally aim to prevent DAMP release, neutralize extracellular DAMPs, or inhibit downstream receptor-mediated signaling pathways [159]. Representative approaches include HMGB1 neutralization, inhibition of P2X7 receptor axis [159,191], modulation of extracellular ATP metabolism via the CD39/CD73 pathway [192], blockade of TLRs [60] and RAGE signaling [193], and inhibition of inflammasome-related pathways, particularly NLRP3 and IL-1β signaling [194].

Among DAMPs, HSPs and particularly HSP90 have attracted considerable interest because of its dual role in maintaining tumor cell survival, stabilizing oncogenic signaling networks, and regulating inflammatory responses [195]. Although HSP inhibitors have demonstrated limited efficacy as monotherapies, growing evidence suggests that combining HSP-targeting strategies with immunotherapy or agents that modulate inflammatory signaling may enhance antitumor responses and improve therapeutic outcomes [196].

Overall, targeting DAMPs and their downstream signaling pathways represents a promising therapeutic framework in mesothelioma, with the potential to simultaneously address chronic inflammation, immune dysregulation, and tumor cell survival mechanisms.

#### 5.2.1. HMGB1 Neutralization

Persistent extracellular HMGB1 drives inflammasome activation, IL-1β secretion, and the maintenance of a chronic inflammatory microenvironment that favors tumor development. Consistent with this role, pharmacological inhibition of HMGB1 signaling using ethyl pyruvate significantly reduced asbestos-induced inflammation and delayed mesothelial transformation in preclinical models, providing evidence for HMGB1-targeted therapeutic strategies in malignant mesothelioma [197].

Among HMGB1-targeting approaches, BoxA has shown encouraging activity in preclinical mesothelioma models. Treatment with BoxA significantly reduced tumor growth in animal models, as demonstrated by high-frequency ultrasound and optical imaging analyses [198]. Mechanistically, BoxA binds CXCR4 and promotes CXCR4-CD47 co-internalization, thereby enhancing macrophage-mediated phagocytosis of tumor cells and facilitating subsequent T-cell priming. This process triggers adaptive antitumor immunity through a mechanism termed Immunogenic Surrender (IGS) [199]. Although both IGS and ICD can generate antitumor immune responses, they differ substantially in their initiating mechanisms. ICD is a death-driven process in which stressed or dying tumor cells expose and release DAMPs that activate antigen-presenting cells and stimulate tumor-specific T-cell responses [200,201]. In contrast, IGS is a clearance-driven mechanism that does not require a specific immunogenic death program; instead, tumor cells become susceptible to phagocytic clearance by innate immune cells, leading to antigen presentation and adaptive immune activation [199,202].

In addition, several other HMGB1-targeting strategies have been explored. These include glycyrrhizin, a natural triterpene that binds HMGB1 and inhibits its pro-inflammatory activity; G-quadruplex aptamers capable of interfering with HMGB1-mediated signaling; and salicylates, including aspirin, which can modulate HMGB1 release and downstream inflammatory responses [61,197,203,204,205,206].

Although these approaches remain at the preclinical stage, HMGB1-targeted therapies represent a promising strategy to interrupt the self-perpetuating damage–inflammation cycle that drives mesothelioma initiation, progression, and immune dysregulation. Future studies will be required to determine whether HMGB1 inhibition can be successfully integrated with current treatment modalities, including chemotherapy and immune checkpoint blockade.

#### 5.2.2. RAGE Inhibition

In mesothelioma, RAGE is particularly relevant due to the abundance of its ligands within the asbestos-injured pleural microenvironment. RAGE activation supports tumor cell survival and invasiveness, whereas soluble RAGE (sRAGE) or pharmacological receptor antagonism can attenuate inflammatory signaling and tumor progression [207].

Therapeutically, different strategies targeting the RAGE axis have shown promising anti-inflammatory and antitumor effects in preclinical studies, including small-molecule inhibitors, genetic silencing approaches, and disruption of the RAGE–DIAPH1 interaction [193,208,209]. Pharmacological inhibition of RAGE via neutralizing antibody reduced mesothelioma cell viability and proliferation, particularly in cells expressing a high level of HMGB1 [61]. Because RAGE acts as a convergent receptor for multiple DAMP ligands, its inhibition may overcome the redundancy that limits single-cytokine targeting strategies.

Importantly, RAGE modulation may also synergize with immunotherapy. DAMP-driven signaling contributes to macrophage polarization, myeloid cell recruitment, T-cell dysfunction, and immune checkpoint regulation, suggesting that targeting the HMGB1–RAGE axis could enhance antitumor immune responses while limiting chronic tumor-promoting inflammation [70]. However, given the context-dependent role of DAMP signaling in both immune activation and tumor progression, effective therapeutic approaches may require temporal and combinatorial strategies.

An additional regulatory layer is provided by sRAGE—a circulating isoform lacking transmembrane and intracellular domains—which functions as a decoy receptor. By binding extracellular DAMPs, sRAGE prevents their interaction with membrane-bound RAGE, and attenuates downstream inflammatory signaling cascades [210,211,212], exerting anti-inflammatory activity in tissue regeneration [213]. In mesothelioma, modulating the balance between membrane-bound RAGE and sRAGE may therefore influence both inflammatory tone and therapeutic responsiveness. Overall, RAGE inhibition represents a promising strategy to disrupt DAMP-driven amplification loops in mesothelioma.

#### 5.2.3. TLR Blockade

TLRs, particularly TLR2, TLR4, and TLR9, are activated by asbestos-induced DAMPs and contribute to the establishment of chronic NF-κB-driven inflammatory signaling [149]. While TLR signaling is essential for host defense during acute infection, its persistent activation in cancer contexts can instead promote tumor progression, immune evasion, and therapeutic resistance [214]. In mesothelioma, continuous stimulation of TLR pathways by DAMPs such as HMGB1, HSPs, and extracellular nucleic acids sustains the production of pro-inflammatory cytokines, including IL-1β, IL-6, and TNF-α, thereby reinforcing a tumor-supportive microenvironment [60]. Accordingly, modulation of TLR signaling has been explored as a therapeutic strategy to attenuate chronic inflammation and enhance antitumor immune responses in mesothelioma [215].

Preclinical evidence supports TLRs, particularly TLR4, as potential therapeutic targets in malignant mesothelioma. Pharmacological inhibition of TLR4 with TAK-242 suppresses mesothelioma cell proliferation, migration, and invasion, while reducing HMGB1 expression, suggesting an HMGB1–TLR4 signaling loop that contributes to tumor-promoting inflammation [60]. In parallel, CUL4B (Cullin 4B), a component of the CRL4B E3 ubiquitin ligase complex, may regulate HMGB1-mediated signaling through epigenetic control of HDAC activity. In pleural mesothelioma, HMGB1 hyperacetylation promotes its cytoplasmic translocation and secretion, enabling extracellular HMGB1 to activate TLR2/TLR4 and sustain chronic inflammation. Collectively, these findings highlight the CUL4B–HDAC–HMGB1–TLR axis as a potential therapeutic target to disrupt tumor-promoting inflammatory signaling in pleural mesothelioma [216].

#### 5.2.4. Inflammasome Modulation

The NLRP3 inflammasome can be triggered by multiple asbestos-induced danger signals, including HMGB1, extracellular ATP, mitochondrial dysfunction, and pro-inflammatory cytokines [110,217]. Therapeutic strategies targeting the NLRP3 pathway—including NLRP3 itself, caspase-1, and IL-1 signaling—are being actively investigated to reduce chronic inflammation and enhance responses to anticancer therapies [194,218]. Among NLRP3-targeting compounds, MCC950 is the most widely studied selective inhibitor and has demonstrated significant anti-inflammatory and antitumor effects in preclinical models [219,220,221]. Although most pharmacological inhibitors of NLRP3—such as colchicine, tranilast, and oridonin—have been primarily investigated in inflammatory and cardiovascular diseases, their mechanisms of action provide a strong rationale for evaluating inflammasome-targeted strategies in mesothelioma and other inflammation-driven malignancies [222,223,224,225].

#### 5.2.5. DAMP Signaling and Immune Checkpoint Inhibitor Response

Immune checkpoint inhibitors (ICIs) have reshaped the therapeutic landscape of mesothelioma. The phase III CheckMate 743 trial demonstrated a significant survival benefit with first-line nivolumab plus ipilimumab compared with platinum-based chemotherapy in unresectable pleural mesothelioma, establishing dual PD-1 and CTLA-4 blockade as a standard therapeutic strategy [1]. These findings highlight the importance of identifying molecular mechanisms and biomarkers that influence ICI response and resistance.

Recently, DNA methylation-defined mesothelioma subtypes with distinct immune profiles were associated with ICI response and survival, highlighting the potential of molecular and immune features to predict immunotherapy outcomes [226].

An emerging question is whether DAMP signaling may influence sensitivity or resistance to ICIs. Effective checkpoint blockade requires pre-existing or inducible tumor-specific T-cell immunity, and DAMPs released or exposed during ICD can contribute to this process. In mesothelioma cells, induction of ICD has been associated with CALR surface exposure together with extracellular ATP and HMGB1 release, indicating that immunogenic DAMP signaling is functional [227].

Moreover, the HMGB1–TLR4 axis provides a link between tumor-cell death and adaptive immunity. In other tumor models, HMGB1 released from dying cancer cells engages TLR4 on dendritic cells and promotes efficient antigen processing and antitumor T-cell responses following chemotherapy or radiotherapy [228]. Moreover, experimental induction of HMGB1-dependent innate sensing through TLR4–MyD88 signaling can enhance tumor-specific CD8+ T-cell responses and sensitize poorly responsive tumors to PD-1/PD-L1 blockade [229]. These observations provide a rationale for combining therapies that promote immunogenic DAMP release with ICIs.

RAGE signaling may also influence the efficacy of ICIs. In colorectal adenomas, activation of the S100A11–RAGE axis promotes immune evasion, whereas targeting RAGE enhances the antitumor efficacy of anti-PD-1 therapy, supporting a functional interaction between RAGE signaling and checkpoint resistance [230]. Consistently, in NSCLC, higher AGEs and RAGE levels negatively influenced therapeutic efficacy of PD-1 inhibitor treatment [231]. S100A9 and HMGB1 were shown to promote the generation of MDSCs in melanoma through TLR4 and, to a lesser extent, RAGE signaling, suggesting that targeting these pathways may alleviate myeloid-mediated immunosuppression and improve ICI efficacy [232].

Overall, these findings suggest that DAMP signaling may modulate ICI response. CALR exposure, HMGB1 release, TLR4, and RAGE signaling may contribute to this process by shaping immune activation in the TME. However, their relevance to ICI response remains largely unexplored in mesothelioma, and no DAMP has yet been validated as a predictive biomarker, so further studies linking DAMP profiles to immune features and clinical outcomes are needed.

## 6. Open Questions and Future Directions

Despite increasing evidence supporting the involvement of DAMPs in mesothelioma, several key questions remain unresolved. A central challenge is understanding how chronic asbestos-induced sterile inflammation transitions from protective to tumor-promoting response. In particular, the temporal dynamics of DAMP signaling remain poorly defined. While transient DAMP release may support immune surveillance and tissue repair, persistent exposure to molecules such as HMGB1, ATP, mitochondrial DNA, and HSPs appears to sustain chronic inflammation, fibrosis, and immune dysfunction. Defining the molecular and temporal switches governing this transition could reveal opportunities for early intervention.

Another major gap concerns the biological heterogeneity of individual DAMPs. HMGB1 is one of the most extensively studied DAMP in mesothelioma and a promising diagnostic and prognostic biomarker; however, the specific contribution of its redox isoforms to inflammation, autophagy, immune modulation, and tumor progression remains incompletely understood [12,42,43,44,45,61]. Similarly, the roles of other DAMPs, including extracellular ATP, S100 proteins, mitochondrial DNA, and heat shock proteins, have not been fully elucidated in mesothelioma biology.

From a translational perspective, the development of integrated DAMP-based signatures represents an important unmet need. Most studies have focused on single biomarkers, whereas mesothelioma is driven by a complex and redundant network of inflammatory mediators. Future investigations integrating circulating and pleural DAMP profiles with genomic, metabolic, and immune parameters may improve patient stratification, prognostic accuracy, and prediction of treatment response.

The context-dependent nature of DAMP signaling further complicates its therapeutic exploitation. Although DAMP release can enhance ICD, chronic activation of DAMP-associated pathways promotes inflammasome signaling, cytokine production, and immunosuppressive remodeling of the tumor microenvironment. Therefore, therapeutic strategies should aim to suppress chronic pro-tumor inflammation while preserving beneficial immunogenic signaling [233].

Emerging pathways provide additional opportunities for investigation. Among these, the IL-33/ST2 axis has gained attention for its dual role in regulating inflammation and antitumor immunity [154,155,156].

Despite substantial progress in delineating DAMP-associated pathways, several aspects remain insufficiently understood in mesothelioma. A major limitation is the functional redundancy and context dependency of these networks, as multiple DAMPs activate overlapping receptors and converge on shared downstream pathways, including NF-κB, MAPKs, inflammasomes, and cGAS–STING signaling [152]. This redundancy may limit the efficacy of single-target therapeutic strategies. Moreover, the dual role of DAMP signaling—capable of promoting both tumor progression and antitumor immunity—further complicates therapeutic exploitation [61,228,234]. In mesothelioma specifically, the balance between chronic inflammation and immunogenic cell death remains poorly defined.

Future studies integrating spatiotemporal profiling of DAMP release with single-cell transcriptomics, spatial omics, and systems immunology approaches will be essential to resolve this complexity and enable rational combination therapies targeting key nodal points in DAMP signaling while preserving antitumor immunity. Ultimately, advances in mesothelioma treatment are likely to arise from integrated strategies combining immunomodulation, metabolic targeting, and personalized molecular profiling. Defining how DAMP-targeting approaches interact with immune checkpoint blockade, inflammasome inhibition, and emerging epigenetic or metabolic therapies will be crucial for translating mechanistic insights into durable clinical benefit for patients with mesothelioma.

## 7. Conclusions

Mesothelioma illustrates how persistent tissue damage can convert physiological danger signaling into a chronic inflammatory program that favors malignant progression. DAMPs, particularly HMGB1, connect asbestos-induced injury with innate immune activation, tumor–host interactions, and therapeutic responses. Their context-dependent functions also represent the major challenge for clinical translation: future strategies will need to identify which DAMP states promote tumor progression and which support productive antitumor immunity. Resolving this distinction will be essential for developing biologically informed biomarkers and therapeutic approaches in mesothelioma.

## Data Availability

No new data were created or analyzed in this study. Data sharing is not applicable to this article.

## Abbreviations

The following abbreviations are used in this manuscript:

AGE	Advanced Glycation End Products
AKT	Protein kinase B
ATP	Adenosine triphosphate
BAP1	BRCA1-associated protein 1
BCL-xL	B-cell lymphoma—extra large
BRCA1	Breast cancer type 1 susceptibility protein/gene
Ca^2+^	Calcium ion
CAF	Cancer-Associated Fibroblast
CALR	Calreticulin
CD39	Cluster of Differentiation 39
CD47	Cluster of Differentiation 47
CD73	Cluster of Differentiation 73
CD91	Cluster of Differentiation 91
CDF	Cancer-Derived Fibroblast
CDKN2A	Cyclin-Dependent Kinase Inhibitor 2A
cfDNA	Cell-free DNA
cGAS-STING	Cyclic GMP-AMP synthase–stimulator of interferon genes
ctDNA	Circulating tumor DNA
CUL4B	Cullin 4B
CXCL12	C-X-C motif chemokine ligand 12
CXCR4	C-X-C chemokine receptor type 4
DAMP	Damage-associated molecular pattern
DIAPH1	Diaphanous-related formin 1
DNA	Deoxyribonucleic acid
ECM	Extracellular matrix
eIF2a	Eukaryotic initiation factor-2alpha
EMT	Epithelial–Mesenchymal Transition
ER	Endoplasmic Reticulum
ERK	Extracellular signal-Regulated Kinase
EV-RNA	Extracellular Vesicle -RNA
exRNA	Extracellular RNA
GPRC6A	G protein-coupled receptor family C group 6 member A
HDAC1	Histone deacetylase 1
HIF1α	Hypoxia-inducible factor 1-alpha
HMGB1	High-Mobility Group Box 1
HSP	Heat Shock Protein
HSP70	Heat Shock Protein 70
HSP90	Heat Shock Protein 90
ICD	Immunogenic Cell Death
ICI	Immunogenic Checkpoint Inhibitors
IGS	Immunogenic Surrender
IL-18	Interleukin-18
IL-1RAcP	Interleukin-1 receptor accessory protein
IL1RL1	Interleukin-1 Receptor Like 1
IL-1α	Interleukin-1 alpha
IL-1β	Interleukin-1 beta
IL-33	Interleukin-33
IL-6	Interleukin-6
IL-8	Interleukin-8
IRFs	Interferon Regulatory Factors
IRF3	Interferon Regulatory Factor 3
IRF7	Interferon Regulatory Factor 7
JAK	Janus kinase
LATS2	Large Tumor Suppressor Kinase 2
LRP1	Low-Density Lipoprotein Receptor-Related Protein 1
MAPK	Mitogen-Activated Protein Kinase
MDSC	Myeloid-Derived Suppressor Cell
mtDAMP	Mitochondrial Damage-Associated Molecular Pattern
mtDNA	Mitochondrial DNA
MyD88	Myeloid differentiation primary response 88
NF2	Neurofibromin 2
NF-kB	Nuclear factor kappa-light-chain-enhancer of activated B cells
NLR	Nucleotide-binding and oligomerization domain (NOD)-like receptor
NLRP3	NOD-, LRR-, and pyrin domain-containing protein 3
NLS	Nuclear Localization Signal
P2X7	P2X purinoceptor 7
P2Y	P2Y purinergic receptors
P53	Tumor protein p53
PAMP	Pathogen-Associated Molecular Pattern
PERK	Protein kinase R-like endoplasmic reticulum kinase
PI3K	Phosphoinositide 3-Kinase
PRR	Pattern-recognition receptor
RAGE	Receptor for advanced glycation end products
RNA	Ribonucleic Acid
ROS	Reactive Oxygen Species
S100	S100 calcium-binding protein family
SDF-1	Stromal cell-derived factor 1, also known as CXCL12
sRAGE	Soluble Receptor for Advanced Glycation End Products
SRC-FAMILY	SRC family kinases
ST2	Suppression of Tumorigenicity 2
STAT	Signal Transducer and Activator of Transcription
TAM	Tumor-Associated Macrophage
TGF-β	Transforming Growth Factor beta
TLR	Toll-Like Receptor
TLR2	Toll-Like Receptor 2
TLR4	Toll-Like Receptor 4
TLR9	Toll-Like Receptor 9
TME	Tumor microenvironment
TNFα	Tumor Necrosis Factor alpha
TRAP1	Tumor necrosis factor receptor-associated protein 1
TRIF	TIR-domain-containing adapter-inducing interferon-β
